# Enhancing Epilepsy Seizure Detection Through Advanced EEG Preprocessing Techniques and Peak-to-Peak Amplitude Fluctuation Analysis

**DOI:** 10.3390/diagnostics14222525

**Published:** 2024-11-12

**Authors:** Muawiyah A. Bahhah, Eyad Talal Attar

**Affiliations:** 1 Department of Electrical and Computer Engineering, Faculty of Engineering, King Abdulaziz University, Jeddah 21589, Saudi Arabia; mbahhah0001@stu.kau.edu.sa; 2 Center of Excellence in Intelligent Engineering Systems (CEIES), King Abdulaziz University, Jeddah 21589, Saudi Arabia

**Keywords:** epilepsy, seizure, focal epilepsy, brain, independent component analysis (ICA), epoch extraction, ERP, rejection component, event-related potentials, ITC, EEGLAB

## Abstract

**Objectives:** Naturally, there are several challenges, such as muscular artifacts, ocular movements and electrical interferences that depend on precise diagnosis and classification, which hamper exact epileptic seizure detection. This study has been conducted to improve seizure detection accuracy in epilepsy patients using an advanced preprocessing technique that could remove such noxious artifacts. **Methods:** In the frame of this paper, the core tool in the area of epilepsy, EEG, will be applied to record and analyze the electrical patterns of the brain. The dataset includes recordings of seven epilepsy patients taken by the Unit of Neurology and Neurophysiology, University of Siena. The preprocessing techniques employed include advanced artifact removal and signal enhancement methods. We introduced Peak-to-Peak Amplitude Fluctuation (PPAF) to assess amplitude variability within Event-Related Potential (ERP) waveforms. This approach was applied to data from patients experiencing 3–5 seizures, categorized into three distinct groups. **Results:** The results indicated that the frontal and parietal regions, particularly the electrode areas Cz, Pz and Fp2, are the main contributors to epileptic seizures. Additionally, the implementation of the PPAF metric enhanced the effectiveness of seizure detection and classification algorithms, achieving accuracy rates of 99%, 98% and 95% for datasets with three, four and five seizures, respectively. **Conclusions:** The present research extends the epilepsy diagnosis with clues on brain activity during seizures and further demonstrates the effectiveness of advanced preprocessing techniques. The introduction of PPAF as a metric could have promising potential in improving both the accuracy and reliability of epilepsy seizure detection algorithms. These observations provide important implications for control and treatment both in focal and in generalized epilepsy.

## 1. Introduction

Epilepsy is a neurological disorder caused by abnormal electrical activity in the brain. Such disorder leads to seizures. Thus, generally, about 50 million people are affected with epilepsy worldwide, making it one of the most common neurological disorders [1]. The epidemiology of epilepsy is highly variable; the prevalence rates range from 0.5% to 1% of the world population. It can be more often diagnosed among children and the elderly [2].

The etiology of epilepsy is complex and generally considered as multifactorial, involving both genetic and structural and environmental factors. Genetic predispositions are strong, with many epilepsy syndromes tied to specific genetic mutations [3].

Cortical malformations, other structural abnormalities and acquired ones such as those due to brain injury or infection, also constitute important causes for the development of epilepsy [4]. Identification of such underlying mechanisms will, in turn, provide the basis for efficient diagnostic and therapeutic strategies.

Neurophysiologically, epilepsy has been attributed to some dysfunction of the brain, resulting in abnormal electrical discharges that further cause generalized or focal seizures. In generalized seizures, wide regions of the brain are involved, while the focal seizure originates from a particular area of the brain [5]. Seizures reflect the dynamic electrical activity of the brain that can be tapped by electroencephalography, or EEG, which is an important tool in epilepsy diagnosis and management [3].

The purpose of this study was to perform seizure analyses on EEG recordings, both ictal and interictal. Such data need to be annotated as to whether the EEG was recorded during a tonic or phasic seizure and if the seizure was generalized or focal. For correct analysis and interpretation of an EEG recording, it is quite important clinically to know such information [4]. For example, generalized seizures involve generalized electrical discharges throughout the brain, whereas focal seizures originate from a specific area of the brain [5]. Such differences may drastically affect the analysis of EEG patterns and the effectiveness of diagnostic methods.

Despite its critical role, traditional EEG methods face limitations, including sensitivity to artifacts and variability in seizure detection [1]. Recent advancements in EEG analysis, such as machine learning algorithms and novel signal processing techniques, aim to address these challenges and enhance diagnostic accuracy [2,3]. However, issues persist with EEG diagnostics, such as the misinterpretation of EEG patterns and interference from artifacts [6,7,8,9].

This paper has tried to address these lacunae by proposing a novel epilepsy diagnosis methodology, integrating state-of-the-art computational techniques along with EEG analysis. The proposed approach will increase seizure detection accuracy and reduce diagnostic errors in the field of epilepsy diagnostics to further clinically effective practices. It will provide a more reliable approach toward the identification of epileptic patterns and improvement of overall patient outcomes.

## 2. Methods

### 2.1. Data Collection

In the current study, the analysis consists of EEG recordings from seven epileptic patients. These recordings were acquired through the Unit of Neurology and Neurophysiology at the University of Siena, Italy. The recordings were performed under the PANACEE project related to describing, detecting and predicting epileptic seizures and wake–sleep transitions [10].

The PANACEE project contributes to epilepsy research by leveraging EEG data to detect seizures and wake–sleep transitions. Its focus is on improving seizure detection accuracy and understanding the neurophysiological patterns associated with these transitions, which are crucial for enhancing patient monitoring and treatment [11]. In this project, patients were admitted for pre-surgical evaluation to determine the nature and localization of their seizures. The recording sessions were carried out with standard video-EEG monitoring, in which each patient was continuously monitored over quite a long period of time in order to also cover awake and sleep states [11].

The EEG recordings were carried out on a 29-channel EEG machine by placing electrodes according to the International 10–20 system. Sampling was performed at high temporal resolutions, usually in the range of 200–500 Hz, to capture the fine details of seizure activity. A 0.5 Hz high-pass filter and a 70 Hz low-pass filter were applied to eliminate muscle and other non-neural artifacts from the signal [11].

Anticonvulsant medications, when clinically appropriate, were tapered or withheld for patients in advance of the recordings to increase the likelihood that seizure events would be recorded during the period of monitoring.

This procedure ensured the best possible conditions for identifying spontaneous seizures in a controlled environment [11].

The recordings included both the awake and sleep states, as seizure activity often correlates with changes in the brain’s state of arousal. Capturing both states allowed for a more comprehensive assessment of seizure dynamics and wake–sleep transitions [11].

The study was completed within the frames of the PANACEE project, from January 2020 to December 2023. This period saw the patients in continuous video-EEG monitoring for pre-surgical evaluation, thus allowing extensive seizure data acquisition and analyzing wake–sleep transitions in relation to epilepsy.

The study involved patients aged 18–75 years of age who had a confirmed diagnosis of drug-resistant focal epilepsy and thus were candidates for pre-surgical evaluation. They should have had at least 3–4 seizures per month to ensure adequate seizure data during the EEG monitoring period. All these patients had to be able to undergo continuous video-EEG monitoring that captured both awake and sleep states for seizure detection. The other criterion for selection included patients with a need for pre-surgical investigation to determine the feasibility of epilepsy surgery. This was particularly among seizures localized to one side of the hemisphere. The last one was that all participants had to give informed consent, or their guardians had to consent on their behalf.

Patients with primary generalized epilepsy were also excluded, as the interest was restricted to those patients who suffered from focal epilepsy. Several other comorbid conditions include active psychiatric disorders, severe cardiovascular conditions and neurodegenerative diseases that may interfere with EEG monitoring or affect the outcomes of the study. Pregnant women were not included, given the risks associated with medication adjustments and prolonged monitoring. Patients who were unable or unwilling to taper off anti-seizure medications according to the protocol, or who had a history of poor compliance in similar studies, were excluded as well. Additionally, individuals with metallic implants (such as pacemakers or neurostimulators) that could interfere with EEG recording quality were not included. Finally, patients who could not provide informed consent due to cognitive impairment or legal restrictions were excluded from the study [12].

The study specifically analyzed data from seven individuals, each of whom experienced 3–5 seizures, as detailed in Table 1. EEG recordings were obtained during both ictal (seizure) and interictal (non-seizure) periods to provide a comprehensive view of brain activity.

### 2.2. Clinical Details of Seizures

Tonic seizures, which involve sustained muscle contraction, and phasic seizures, which involve jerking of muscles, were included to give some clarity on clinical matters in the EEG recording. The seizures were categorized as either generalized or focal, that is, influencing large parts or specific parts of the brain. This variation allowed for a thorough examination of EEG patterns associated with different seizure types and their impact on brain activity.

### 2.3. Ethical Approval

The study presented here was approved by the Ethical Committee of the University of Siena. Following the Declaration of Helsinki, written informed consent was obtained from each subject. Written consent concerning video recording and the use of data for scientific purposes was given by each patient [10].

### 2.4. EEG Processing with EEGLAB

EEGLAB, a widely used software for EEG data analysis, was employed for preprocessing and analyzing the EEG data [12]. The processing pipeline included the following steps:

#### 2.4.1. Data Preprocessing

Initial preprocessing involved loading the raw EEG data into EEGLAB. This step included filtering to remove noise, epoch extraction to segment the data around specific events, baseline removal to normalize the data and resampling to ensure uniform data intervals. Time-locked data epochs were extracted to focus on specific experimental events [13].

Certain channels were excluded during data loading, and a combination of high- and low-pass filters was applied. Independent Component Analysis (ICA) was then performed, preparing the data for component rejection. The study utilized automated techniques like Clean Raw Data (CRD) and Artifact Subspace Reconstruction (ASR) to detect and eliminate artifacts, ensuring data purity [14]. The interictal EEG data were analyzed using time-locked epoch extraction and Independent Component Analysis (ICA).

#### 2.4.2. Re-Referencing and Filtering

Re-referencing was performed using the Common Average Reference (CAR) method, which enhances signal quality by reducing noise and isolating cerebral activity. High-pass and low-pass filters were applied to eliminate slow drifts and improve signal clarity. The filter parameters were optimized through trial and error to achieve the best results for EEG signal processing [15].

#### 2.4.3. Data Structures and Events

EEGLAB organizes data into a single structure (EEG), which includes information on data acquisition parameters, events, channel locations and epoch details. This structure facilitated subsequent analyses, including data epoch extraction, trial sorting and the creation of Event-Related Potential (ERP) images [16]. Channel ERP images and graphical depictions of Event-Related Potentials (ERPs) derived from EEG data were computed for each channel.

The identification of significant ERP components, particularly in the frontal and parietal regions, was crucial for understanding neural activity associated with epilepsy seizures involving four and five seizures [17].

#### 2.4.4. Peak-to-Peak Amplitude Fluctuation (PPAF)

The PPAF is a measure of fluctuation from which the amplitude of successive peak-to-peak variability in the EEG signal is calculated to study the fluctuations. The fundamental idea behind the use of PPAF lies in its computation of the difference in the peak-to-peak amplitude of the EEG signal because this region indicates greater electrical activity corresponding to seizures [18].

The general equation for calculating Peak-to-Peak Amplitude Fluctuation is as follows [18]:

(1) $$PPAF=\frac{1}{N}\sum_{i=1}^{N}\left({Peak}_{i}-{Trough}_{i}\right)$$

where

- ${Peak}_{i}$ represents the amplitude of the i-th peak in the EEG signal;
- ${Trough}_{i}$ represents the amplitude of the i-th trough following the i-th peak;
- N is the total number of peak-to-trough pairs.

#### 2.4.5. Visualizing and Analyzing the Data

**Figure 1:**
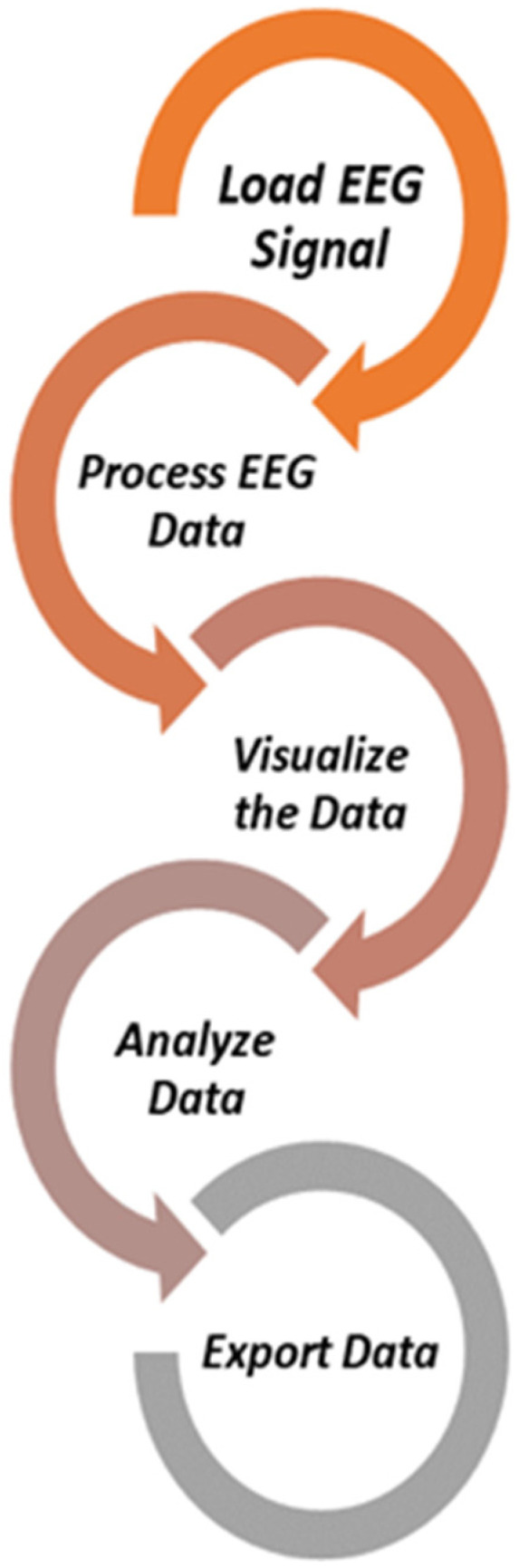
EEG processing stages in the study.

The preprocessed EEG data were visualized and analyzed using EEGLAB’s tools. Independent Component Analysis (ICA) was performed to identify and separate different sources of brain activity, allowing for a more accurate interpretation of EEG patterns [19]. This analysis provided insights into the brain’s electrical activity during both ictal and interictal periods. Figure 1 illustrates the processing stages involved in the study.

## 3. Results

**Figure 2:**
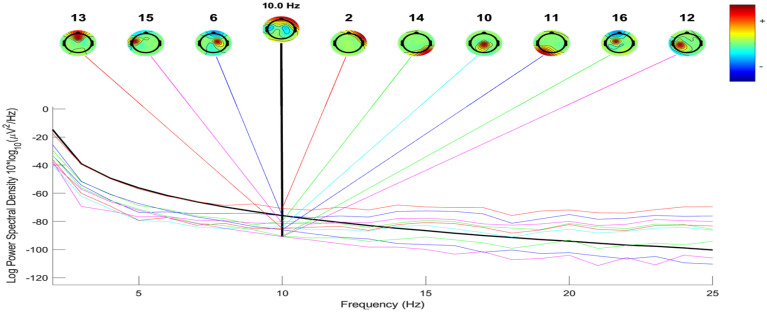
EEG real (−, time acquisition: Power spectrum envelopes and ERP activity of eight most significant independent components.

**Figure 3:**
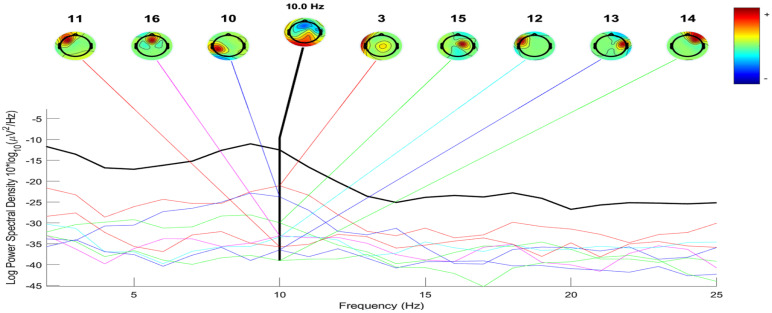
Epilepsy detection for patient with four seizures by examining the IC ERP envelope plots with topographical maps.

**Figure 4:**
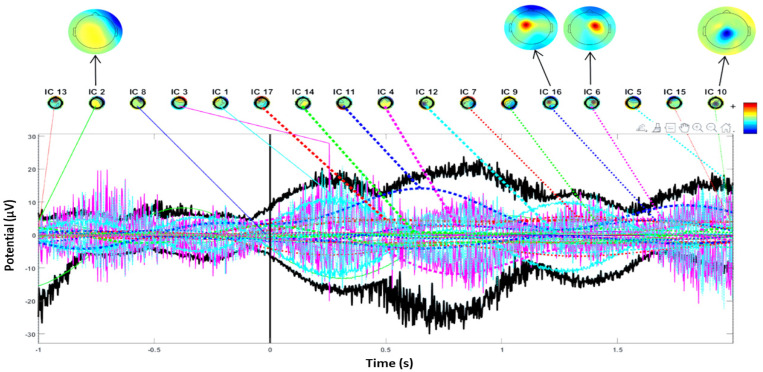
This figure displays the power spectrum and ERP activity of the most significant independent components. The black traces represent ERP envelopes, with key IC contributions highlighted in color. The scalp topography maps illustrate the spatial distribution of the ICs during seizure events.

**Figure 5:**
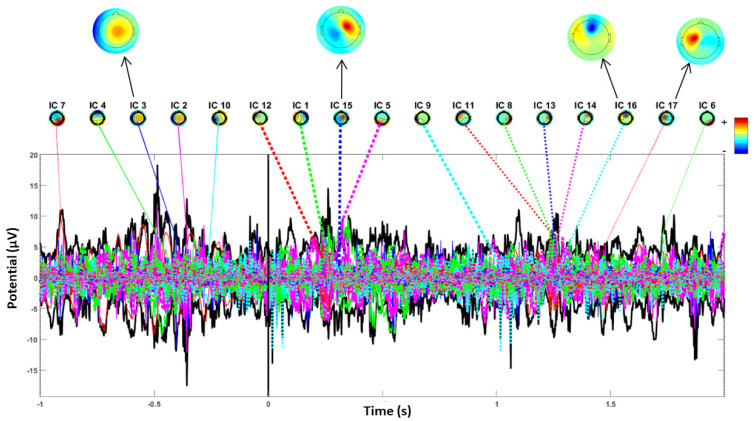
The ERP and power spectrum for a patient with five seizures. Key independent components (IC 3, 15, 16 and 17) are represented along with their spatial projections.

The process of constructing ERP image plots is illustrated in Figure 2, Figure 3, Figure 4 and Figure 5. These plots represent instantaneous trial potentials as colored horizontal lines, accompanied by the average spectrum of the signal and a scalp topography. This methodology effectively identifies and eliminates artifacts such as eye, muscle and line noise.

Figure 2 and Figure 3 provide insight into the EEG signal bandwidth during seizure activities, which occurs in the frequency range of 3–30 Hz. Figure 4 and Figure 5 present the power spectrum of a specific channel, along with the activity spectra of the projection of each of the 29 components onto this channel.

Additionally, scalp power maps of the four most contributing components (2, 6, 10, 16) are displayed for four seizures in Figure 4. In contrast, Figure 5 highlights the largest component (3, 15, 16, 17) for five seizures. Envelopes in Figure 4 and Figure 5 demonstrate the min and max values over all channels at each time point for the five independent components making the largest potential contributions to the ERP.

The black thick traces represent the envelope of the ERP data across all channels, while the thin traces depict the envelopes of the component contributions.

**Figure 6:**
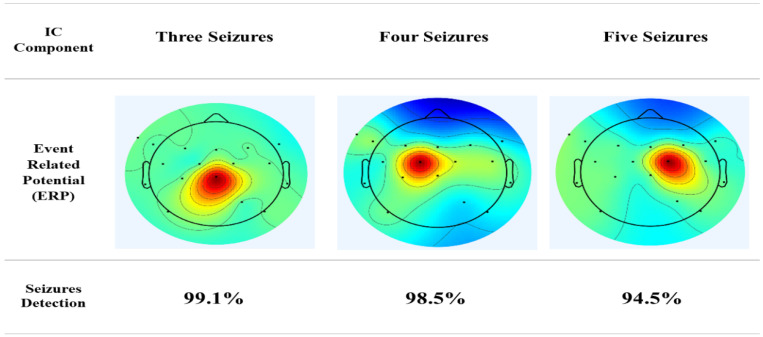
Mean PPAF values for different seizure categories. The percentage of amplitude fluctuations indicates seizure waveform stability across the 3-, 4- and 5-seizure datasets.

The Peak-to-Peak Amplitude Fluctuation (PPAF) percentage, a quantitative assessment of amplitude variability in the ERP waveform, was calculated. The study achieved PPAF percentages of 95.16% and 96.33% for subjects with 4-Record and 5-Record, respectively, as illustrated in Figure 6 This metric serves as an important indicator of ERP waveform stability across participants. Picard’s innovative use of the L-BFGS algorithm in ICA problem-solving was a notable feature, ensuring effective artifact detection and removal [20].

## 4. Discussion

The accurate detection and classification of epilepsy are pivotal for effective patient management, encompassing not only the identification of epileptic convulsions but also the understanding of their underlying neural dynamics. This study utilized advanced preprocessing techniques and introduced the Peak-to-Peak Amplitude Fluctuation (PPAF) metric to enhance the detection and classification of epileptic seizures from EEG data. Table 2 compares the Peak-to-Peak Amplitude Fluctuation (PPAF) method with traditional methods for identifying seizure locations.

EEG remains a cornerstone in epilepsy research due to its ability to capture the brain’s electrical activity non-invasively [21]. However, challenges such as muscle artifacts, ocular movements and electrical noise can compromise the accuracy of seizure detection algorithms [7]. To address these issues, this study employed sophisticated preprocessing techniques to eliminate artifacts and enhance signal quality. The use of EEGLAB software allowed for effective artifact detection and removal, crucial for ensuring the purity of the EEG signal [12].

The preprocessing steps included filtering, epoch extraction, baseline removal and re-referencing, which collectively contributed to improved signal clarity and artifact reduction. Figure 2 and Figure 3 illustrate the frequency bandwidth during seizure activities, aligning with the expected range of 3–30 Hz and highlight the role of specific frequency bands in seizure dynamics [21]. 

The topographical plots revealed significant alterations in the alpha and theta bands, suggesting changes in neural synchronization or hyperactivity during seizures [22,23].

The results of this study underscore the critical role of the frontal and parietal regions in seizure activity. Notably, electrodes Cz, Pz and Fp2 showed substantial involvement during seizure events. This finding aligns with the existing literature, which emphasizes that these brain areas are integral to the generation and propagation of seizure activity. The significant electrical activity detected at these electrode sites suggests that the frontal and parietal regions may contribute to the clinical manifestations observed during seizures, highlighting their importance in understanding seizure dynamics and potential therapeutic targets.

Furthermore, the data suggest that monitoring these specific regions could enhance the accuracy of seizure prediction models, thereby improving patient outcomes through timely interventions. These insights align with previous studies, including Spencer (2002), which discussed the relevance of neural network applications in epilepsy and the potential for more nuanced analyses of seizure localization and characteristics [24,25].

The power spectrum and scalp topography analyses (Figure 4 and Figure 5) provided detailed insights into the frequency characteristics and spatial distribution of seizure-related activity.

Figure 4 and Figure 5 further demonstrated the effectiveness of ICA in isolating significant independent components and their contributions to ERP waveforms. The PPAF percentages reinforce the reliability and stability of the observed EEG patterns during seizures, highlighting the robustness of the proposed method [26,27]. The consistent performance across different seizure scenarios validates the PPAF metric’s utility in capturing characteristic ERP waveforms associated with epileptic events.

The analysis of the provided EEG recordings, as depicted in Figure 4 and Figure 5, reveals critical spike-and-wave patterns that are typically associated with seizure activity in epilepsy [22,28]. These patterns are characterized by sharp spikes followed by slower waveforms, which are indicative of ictal activity. In the EEG data, segments show significant spikes and fluctuations in amplitude, aligning with the spike-and-wave characteristics commonly observed during seizures [29].

In Figure 4, several independent components (e.g., IC 3, IC 4) exhibit varying levels of activity that correlate with these distinctive spike-and-wave patterns. The pronounced spikes observed in these components suggest periods of ictal activity, providing crucial insights into the timing and location of seizures. The topographic maps accompanying these segments illustrate the spatial distribution of electrical activity across the scalp, with specific components such as IC 13 and IC 2 showing activation patterns consistent with seizure-related abnormalities [30].

Conversely, Figure 5 presents recordings from comparable timeframes but lacks the pronounced spike-and-wave patterns observed in the previous figure. This absence may indicate differences in seizure characteristics or a lack of ictal activity during those periods. The independent components analyzed, including IC 7 and IC 4, show variations in amplitude and distribution, but without clear spike-and-wave formations.

The introduction of the PPAF metric as shown in Figure 6 proved to be a robust tool for quantifying amplitude variability in ERP waveforms, achieving high accuracy rates of 95.16% and 96.33% for 4-seizure and 5-seizure datasets, respectively [31].

These findings highlight the effectiveness of our method in detecting and visualizing spike-and-wave patterns, which is crucial for accurate epilepsy diagnosis and management [32]. The ability to identify these patterns, or their absence, enhances the reliability of our approach compared to traditional methods [33,34]. Our advanced preprocessing and analysis techniques contribute to a more precise understanding of seizure dynamics, potentially leading to improved diagnostic and treatment strategies for epilepsy. Continued analysis and monitoring are recommended to further refine detection accuracy and elucidate the underlying neural mechanisms.

The study’s findings have important clinical implications for epilepsy management. Accurate seizure detection and classification are critical for timely intervention and treatment planning. The use of advanced preprocessing techniques and the PPAF metric can improve the precision of seizure detection algorithms, potentially leading to better patient outcomes [35,36].

The focus on frontal and parietal electrode areas aligns with previous research highlighting the significance of these regions in seizure activity [37].

The adaptability of the EEGLAB-based approach to diverse physiological profiles underscores its potential for personalized epilepsy management. Future research could explore the integration of advanced neuroimaging techniques to complement EEG data, enhancing our understanding of the spatiotemporal dynamics of seizures [35,36]. Additionally, addressing challenges such as artifact variability and developing user-friendly wearable devices could further advance epilepsy detection and treatment [36,38]. While the current study demonstrates the utility of advanced preprocessing techniques and the PPAF metric in detecting and classifying focal seizures, the non-inclusion of patients with generalized epilepsy limits the generalizability of these findings. Generalized seizures, which involve widespread brain activity, could present different patterns in EEG data that might also benefit from these methods. Future studies should include patients with generalized epilepsy to assess the applicability of PPAF and other metrics in distinguishing between seizure types.

The study provides valuable information on the detection of epileptic seizures based on EEG; however, some limitations must be considered. Firstly, the sample size in this study was seven patients, possibly limiting the generalization of our results to a larger population. Future studies should be designed to include a larger cohort with the view of improving the generalizability of such findings. Secondly, although the Peak-to-Peak Amplitude Fluctuation was used as one metric of seizure detection in the analysis of this study, we do realize that seizures can manifest under many other patterns and dynamics in EEGs. The use of other metrics, such as spectral power analyses or time–frequency decompositions, may yield a far more complete representation of seizure characteristics and further improve seizure detection accuracy [39]. Each of these limitations minimized or addressed would increase the possible clinical applicability of our findings across diverse populations with epilepsy.

## 5. Conclusions

This study contributes to the field of epilepsy research by introducing advanced preprocessing techniques and the PPAF metric, which collectively enhance the accuracy and reliability of seizure detection algorithms. The findings underscore the importance of frontal and parietal electrode areas and highlight the potential for improved patient management through enhanced diagnostic methods. As the field progresses, ongoing research and technological advancements will continue to shape the future of epilepsy detection and treatment.

## Figures and Tables

**Table 1:** Patient Information.

Patient ID	Age	Gender	Seizure	Localization	Lateralization	EEG Electrodes	Number of Seizure	Recording Time (Seconds)
PN00	55	Male	IAS (Intra-Axial Seizure)	T (Temporal)	R (Right)	29	5	198 s
PN05	51	Female	IAS (Intra-Axial Seizure)	T (Temporal)	L (Left)	29	3	359 s
PN06	36	Male	IAS (Intra-Axial Seizure)	T (Temporal)	L (Left)	29	5	722 s
PN09	27	Female	IAS (Intra-Axial Seizure)	T (Temporal)	L (Left)	29	3	410 s
PN12	71	Male	IAS (Intra-Axial Seizure)	T (Temporal)	L (Left)	29	4	246 s
PN13	34	Female	IAS (Intra-Axial Seizure)	T (Temporal)	L (Left)	29	3	519 s
PN14	49	Male	WIAS (Wide Intra-Axial Sei-zure)	T (Temporal)	L (Left)	29	4	1408 s

**Table 2:** Comparing the Peak-to-Peak Amplitude Fluctuation (PPAF) method with traditional methods for identifying seizure locations.

Criteria	Peak-to-Peak Amplitude Fluctuation (PPAF)	Traditional Methods	References
Accuracy	Higher accuracy in detecting seizure locations	Variable accuracy; dependent on manual inspection or standard algorithms	[3,4]
Sensitivity	Improved sensitivity for identifying seizure events	Moderate sensitivity; may miss subtle seizure activity	[3,4]
Specificity	High specificity; fewer false positives	Varies; potential for higher false positives	[3,4]
Computational Efficiency	Faster processing and analysis	Slower, especially with visual inspection	[3,4]
Ease of Implementation	Easier to implement with automated tools	Requires manual review or standard analysis tools	[3,4]
Clinical Relevance	Provides more precise localization of seizures	Effective but less precise; often requires additional tools	[1,2]

## Data Availability

The data are available online from the open source.
